# Molecular Comparison of Imatinib-Naïve and Resistant Gastrointestinal Stromal Tumors: Differentially Expressed microRNAs and mRNAs

**DOI:** 10.3390/cancers11060882

**Published:** 2019-06-24

**Authors:** Azadeh Amirnasr, Caroline M.M. Gits, Patricia F. van Kuijk, Marcel Smid, Anne L.M. Vriends, Piotr Rutkowski, Raf Sciot, Patrick Schöffski, Maria Debiec-Rychter, Stefan Sleijfer, Erik A. C. Wiemer

**Affiliations:** 1 Department of Medical Oncology, Erasmus MC Cancer Institute, Erasmus University Medical Center, 3015 CN Rotterdam, The Netherlands; a.amirnasr@erasmusmc.nl (A.A.); carolinegits@hotmail.com (C.M.M.G.); p.vankuijk@erasmusmc.nl (P.F.v.K.); m.smid@erasmusmc.nl (M.S.); a.vriends@erasmusmc.nl (A.L.M.V.); s.sleijfer@erasmusmc.nl (S.S.); 2 Department of Soft Tissue/Bone Sarcoma and Melanoma, Marie Skłodowska-Curie Institute-Oncology Center, 02781 Warsaw, Poland; piotr.rutkowski@coi.pl; 3 Department of Pathology, University Hospitals Leuven, Katholieke Universiteit Leuven, 3000 Leuven, Belgium; raf.sciot@uzleuven.be; 4 Department of General Medical Oncology, Leuven Cancer Institute, University Hospitals Leuven, Katholieke Universiteit Leuven, 3000 Leuven, Belgium; patrick.schoffski@uzleuven.be; 5 Research Unit Laboratory of Experimental Oncology, Department of Oncology, Faculty of Medicine, Katholieke Universiteit Leuven, 3000 Leuven, Belgium; 6 Department of Human Genetics, University Hospitals Leuven, Katholieke Universiteit Leuven, 3000 Leuven, Belgium; maria.debiec-rychter@kuleuven.be

**Keywords:** GIST, miRNA, mRNA expression, imatinib, drug resistance, gene networks, IPA pathway analysis

## Abstract

Despite the success of imatinib in advanced gastrointestinal stromal tumor (GIST) patients, 50% of the patients experience resistance within two years of treatment underscoring the need to get better insight into the mechanisms conferring imatinib resistance. Here the microRNA and mRNA expression profiles in primary (imatinib-naïve) and imatinib-resistant GIST were examined. Fifty-three GIST samples harboring primary KIT mutations (exon 9; n = 11/exon 11; n = 41/exon 17; n = 1) and comprising imatinib-naïve (IM-n) (n = 33) and imatinib-resistant (IM-r) (n = 20) tumors, were analyzed. The microRNA expression profiles were determined and from a subset (IM-n, n = 14; IM-r, n = 15) the mRNA expression profile was established. Ingenuity pathway analyses were used to unravel biochemical pathways and gene networks in IM-r GIST. Thirty-five differentially expressed miRNAs between IM-n and IM-r GIST samples were identified. Additionally, miRNAs distinguished IM-r samples with and without secondary KIT mutations. Furthermore 352 aberrantly expressed genes were found in IM-r samples. Pathway and network analyses revealed an association of differentially expressed genes with cell cycle progression and cellular proliferation, thereby implicating genes and pathways involved in imatinib resistance in GIST. Differentially expressed miRNAs and mRNAs between IM-n and IM-r GIST were identified. Bioinformatic analyses provided insight into the genes and biochemical pathways involved in imatinib-resistance and highlighted key genes that may be putative treatment targets.

## 1. Introduction

Gastrointestinal stromal tumors (GISTs) are rare mesenchymal malignancies associated with the gastrointestinal tract that originate from the interstitial cells of Cajal (ICC) or precursors thereof [1]. GISTs and ICC share morphological and immunophenotypic features, notably the expression of KIT and CD34. Molecularly, GISTs are characterized by the presence of oncogenic gain-of-function mutations in *KIT* (~80% of cases) or *PDGFRA* (~10% of cases) [2,3]. *KIT* and *PDGFRA* mutations are absent in the so-called wild-type GISTs (~10% of cases) that may contain mutations in *BRAF*, *NF1*, or defects of the succinate dehydrogenase (SDH) complex [4]. The constitutive activation of KIT and PDFGRA signaling in the majority of GISTs drives tumor growth through the activation of downstream signaling cascades such as the RAS–RAF–MAPK, PI3K–AKT, and STAT3 pathways facilitating cell proliferation and survival [5]. The advent of the tyrosine kinase inhibitor imatinib mesylate, which targets both KIT and PDGFRA, has dramatically improved the outcome of patients with advanced disease [6,7]. Despite this great progress in GIST treatment and the fact that approximately 10% of the patients benefit for more than 10 years from imatinib [8], the majority of patients eventually develop imatinib resistance (acquired resistance) [8] with about 10% of GIST patients experiencing progression already within 6 months of start of therapy (intrinsic resistance) [6,7]. Where in intrinsic resistant cases in particular *KIT* exon 9 mutations or *PDGFRA* D842V mutations are involved [9], acquired resistance may occur because of secondary mutations within *KIT* that interfere with the binding of imatinib [10,11,12,13,14]. These resistance-causing secondary mutations cluster in two regions: (i) ATP-binding pocket (encoded by exons 13 and 14), and (ii) kinase catalytic regions/activation loop (encoded by exons 17 and 18). Such secondary mutations leading to acquired resistance are observed in approximately 50% of GIST patients. The remaining cases with acquired resistance display alternative resistance mechanisms that are much less defined and include *KIT* and *PDFRA* amplification [11,13] and receptor tyrosine kinase switches from KIT to activation of FAK, FYN, or AXL [15,16,17].

A better understanding of the causes yielding imatinib resistance is necessary to improve treatment and outcomes. Here we performed a molecular comparison between a unique set of imatinib-naïve (IM-n) GIST samples (n = 33) and imatinib-resistant (IM-r) GIST samples (n = 20) focusing on microRNA and mRNA expression to reveal molecular pathways associated with imatinib resistance.

## 2. Results

### 2.1. Differentially Expressed microRNAs between Imatinib-Naïve and Imatinib-Resistant GIST Samples 

**Figure 1:**
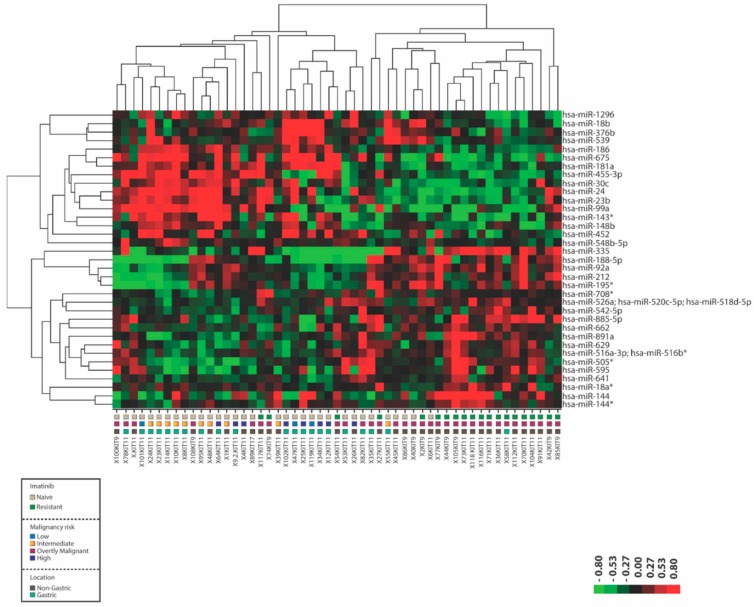
MicroRNA expression distinguishes imatinib-naïve (IM-n) from imatinib-resistant (IM-r) gastrointestinal stromal tumors (GIST). Fresh frozen tumor samples of IM-n and IM-r GIST patients were subjected to miRNA expression profiling. Depicted is the heat map of a supervised hierarchical clustering based on the 35 most significant (*p* < 0.01 and False Discovery Rate (FDR) < 20%) differentially expressed miRNAs. In the heat map red indicates relative high expression and green indicates relative low expression. The colored squares beneath the graph designate IM-n and IM-r samples, the malignancy risk and location of the tumors. Note that the sample codes below also indicate which KIT exon is mutated.

To investigate the molecular events underlying the acquisition of imatinib resistance in GIST we first determined the miRNA expression profiles in fresh frozen IM-n (n = 33) and IM-r (n = 20) GIST samples (Table 1). All imatinib resistant GIST patients displayed resistance after more than 6 months of imatinib treatment implicating acquired resistance mechanisms. Thirty-five significantly (*p* < 0.01 and False Discovery Rate (FDR) <20%) differentially expressed miRNAs were detected between the two groups (Figure 1, Table S1). Figure 1 depicts the heat map from a supervised hierarchical clustering. Two main clusters were discerned, one cluster contained 82% of the IM-n samples and the other cluster included 85% of all IM-r samples. A number of samples of both IM-r and IM-n GISTs were found to miscluster, a fact that could not readily be explained by differences in malignancy risk or tumor location.

**Figure 2:**
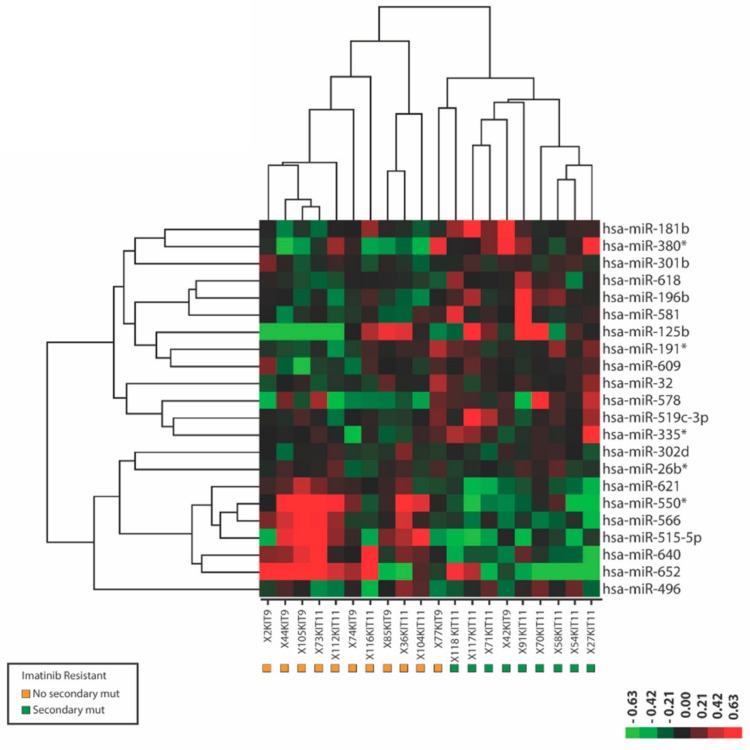
MicroRNAs differentially expressed between imatinib-resistant gastrointestinal stromal tumor (GIST) samples with and without secondary mutations in KIT. Depicted is a heat map of a supervised hierarchical clustering based on the 22 most significant (*p* < 0.01) differentially expressed miRNAs in fresh frozen GIST samples with (green squares) and without (orange squares) secondary imatinib resistance causing mutations in KIT. In the heat map red indicates relative high expression and green indicates relative low expression. Note that the sample codes below also indicate which KIT exon is mutated.

Secondary mutations in *KIT* are a frequent cause of imatinib-resistance in GIST. In the 20 IM-r samples that we analyzed, nine displayed secondary mutations in *KIT* exon 13 (n = 3) and *KIT* exon 17 (n = 6), whereas in the remainder (n = 11) no secondary mutations were observed (Table 1). When we compared the miRNA expression profiles of IM-r samples with and without secondary mutations, we identified 22 miRNAs that were significantly (*p* < 0.01) differentially expressed and almost completely separated the two groups (Figure 2, Table S2). This suggests miRNA biomarker profiles may be associated with the presence/absence of secondary mutations.

### 2.2. mRNA Expression Profiling and Ingenuity Pathway Analyses Reveal Differentially Expressed Genes and Pathways in Imatinib-Naïve and Imatinib-Resistant GIST Samples

**Figure 3:**
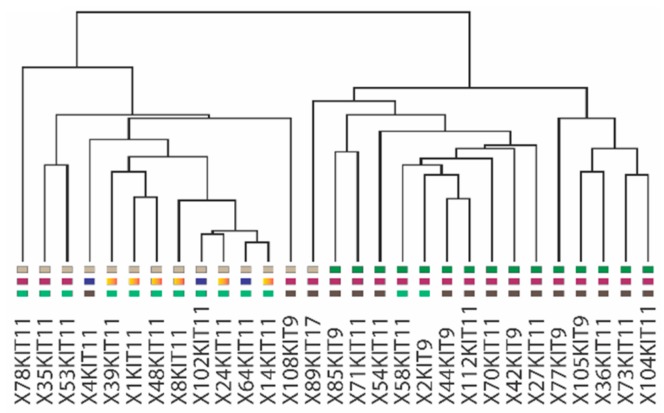
Supervised hierarchical clustering based on differential gene expression discriminates imatinib-naïve (IM-n) and imatinib-resistant (IM-r) gastrointestinal stromal tumor (GIST) samples. Transcript expression profiles were determined using the Affymetrix platform (U133 plus 2) of 29 fresh frozen samples derived of IM-n (n = 14) and IM-r (n = 15) GISTs. Depicted is the cluster tree of a supervised hierarchical clustering based on 352 significant (*p* < 0.008, False Discovery Rate (FDR) < 10%), differentially expressed transcripts. Note that 100% of the IM-r samples are clustered together with a single IM-n GIST sample. The colored squares beneath the graph designate imatinib-naïve and imatinib-resistant samples, the malignancy risk and location of the tumors (see Figure 1). Note that the sample codes below also indicate which KIT exon is mutated.

In order to better understand the genes and molecular pathways involved in imatinib resistance in GIST we performed mRNA expression analyses on a subset (IM-r, n = 15 vs IM-n, n = 14) of our GIST samples. At least 352 genes were identified to be significantly differentially expressed (*p* < 0.008, FDR < 10%) between the two groups (Figure S1; Table S3). Figure 3 shows the cluster tree of a supervised cluster analysis based on the expression of the 352 differentially expressed genes represented by 475 different Affymetrix probe sets (Affymetrix, Santa Clara, CA, USA) (Figure S1). All IM-r samples cluster together as do all IM-n samples except one. A molecular pathway analysis, focusing on canonical pathways and using the Ingenuity platform (Qiagen, Hilden, Germany), was performed with the 352 differentially expressed genes as input. Among these genes, regulators of estrogen-mediated S-phase entry (*p* = 8.29 × 10^−8^), cyclins and cell cycle regulators (*p* = 3.09 × 10^−6^), as well as checkpoint regulators of G2/M DNA damage (*p* = 8.64 × 10^−6^) were overrepresented (Figure S2). Of note, the cyclins A2, B1, B2, D2, and E2, as well as *CDK1* and the E2F transcription factors *E2F7* and *E2F8* were among the most differentially expressed genes found in two or more deregulated pathways (Table S4). Except for *CCND2* (5.4 fold lower in IM-r), all the other seven genes displayed increased expression in IM-r with the fold changes of 2.6 for *CCNA2*, 2.9 for *CCNE2*, 2.3 for *CCNB1*, 2.1 for *CCNB2*, 3.0 for *CDK1*, 2.1 for *E2F7*, and 2.0 for *E2F8* in comparison to the imatinib-naïve setting. 

**Figure 4:**
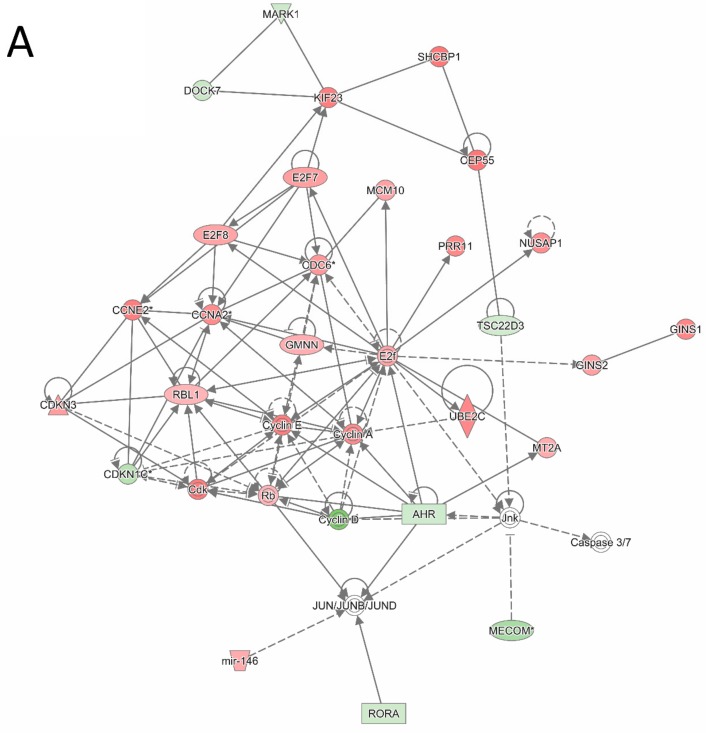

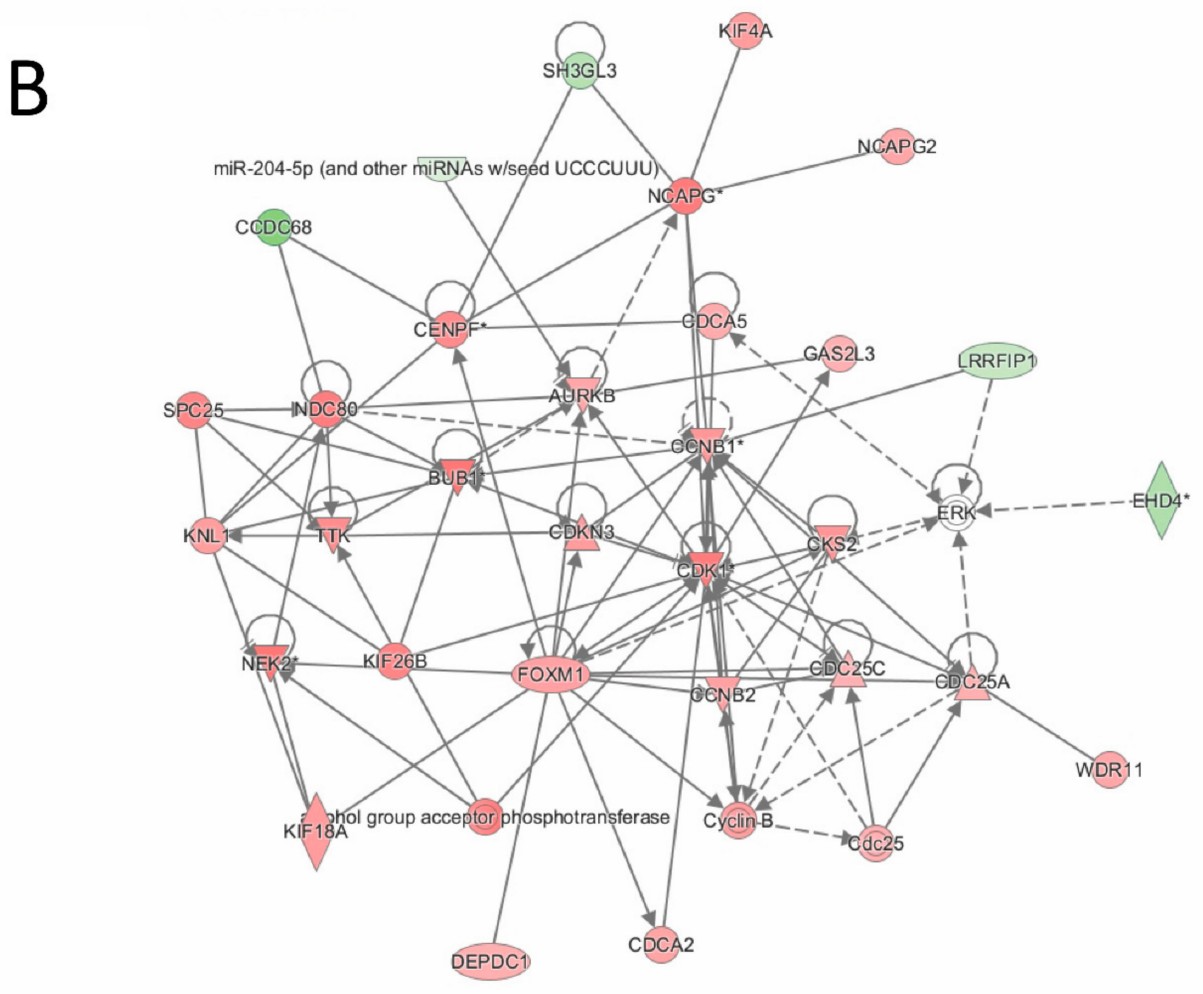
Ingenuity Pathway Analysis indicates the relation between genes differentially expressed between imatinib-naïve (IM-n) and imatinib-resistant (IM-r) gastrointestinal stromal tumor (GIST) samples. The 352 significant differentially expressed genes between IM-r and IM-n GIST samples were used as input for an Ingenuity Pathway Analysis (IPA). The depicted IPA networks illustrate and visualize associations between the genes. (**A**) IPA network highlighting cell cycle related, differentially expressed genes. (**B**) IPA network highlighting CDK1, AURKB, and FOXM1 interactions. Green and red shading indicates relatively low and high expression in the IM-r samples. See Table S5 for an extended symbol legend.

An ingenuity pathway analysis (IPA) was also conducted to examine interactions within the 352 most differentially expressed genes between IM-r and IM-n GIST samples. Figure 4A,B depict two of the largest and most significant interaction networks revealed by IPA (see for a symbol legend Table S5). Figure 4A displays associations between genes involved in cell cycle regulation and consequently cell proliferation. Cyclin A and cyclin E appear as central hubs in the gene network. The interaction network shown in Figure 4B also supports cell cycling and cell proliferation judged by the overexpressed central genes cyclin dependent kinase 1 (CDK1), aurora kinase B (AURKB), and forkhead box protein M1 (FOXM1). CDK1 plays a key role in cell cycle regulation, AURKB regulates the segregation of chromosomes and the spindle checkpoint in mitosis and FOXM1 is a transcription factor essential for cell cycle regulation. 

### 2.3. Integration of Differentially Expressed microRNAs and mRNAs into Networks

Using IPA, we investigated whether interaction networks between mRNAs and miRNAs could be defined to identify and better understand the possible regulatory role of miRNAs-mRNAs interactions in imatinib resistance. To be able to directly compare mRNA with miRNA expression in the same GIST samples, we considered only the differentially expressed miRNAs in the subset of GIST samples that were analyzed by mRNA expression profiling. We identified 88 differentially expressed miRNAs (*p* < 0.03, FDR < 30%) (Table S6). Note that almost 70% of the differentially miRNAs reported in Figure 1 and Table S1 were present in this miRNA selection.

**Figure 5:**
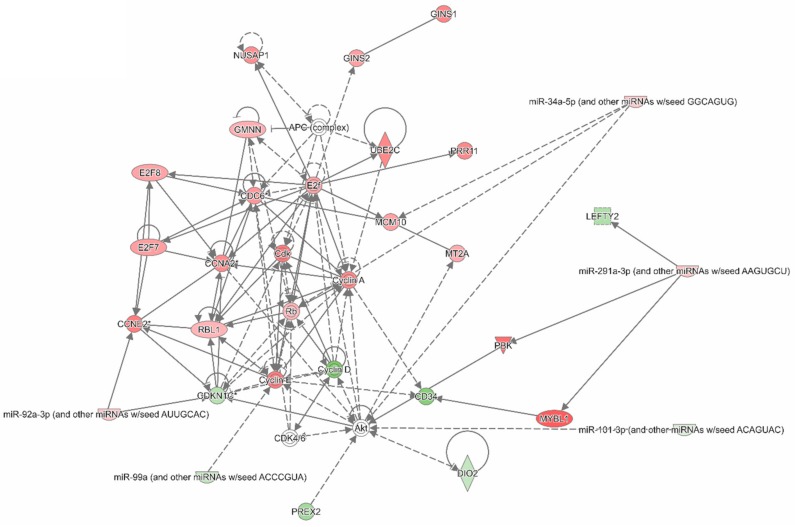
Ingenuity Pathway Analysis integrating differentially expressed genes and microRNAs between imatinib-naïve (IM-n) and imatinib-resistant (IM-r) gastrointestinal stromal tumor (GIST) samples. As input for an Ingenuity Pathway Analysis (IPA) the significantly differentially expressed transcripts (352 genes, *p* < 0.008, False Discovery Rate (FDR) < 10%) and miRNAs (88 miRNAs, *p* < 0.03, FDR < 30%) from the same set of IM-r (n = 15) and IM-n (n = 14) GIST samples were used. The depicted network indicates miRNA–gene interactions relevant in context of the cell cycle. Green and red shading indicates relatively low and high expression in the IM-r samples. See Table S5 for an extended symbol legend.

The gene-miRNA network presented in Figure 5 included most regulatory gene-miRNA interactions and related to cell cycle regulation. The network highlights regulation by miR-92a-3p, miR-99a-5p, and miR-101-3p. The differential expression of selected miRNAs and mRNAs, which were indicated in the text and IPA analyses, were verified by RT-PCR, thereby confirming our findings with the miRNA and mRNA array platforms (Figure S3).

## 3. Discussion

To better understand the mechanisms that account for imatinib resistance, here we molecularly characterized at an mRNA and miRNA level a unique set of IM-n and IM-r GIST samples. Bioinformatic approaches were used to identify signaling pathways and gene networks modulated in imatinib-resistant GISTs.

The reason to look for differentially expressed miRNAs between IM-n and IM-r samples is based on the observations that miRNAs are intimately involved in GIST pathobiology [18,19] and well-known actors in drug resistance mechanisms occurring in cancer types other than GIST [20]. Indeed, we identified miRNAs that distinguished IM-r from IM-n GIST samples. Although the fold changes observed were relatively small, they can still have a significant impact on protein levels because the regulation of multiple targets within the same pathway can amplify their biological effect [21] and different miRNAs may cooperate and have synergistic effects [22]. Previously few other groups studied miRNA expression in relation to imatinib-resistance in GIST as well [23,24]. Akaçakaya et al. compared miRNA expression profiles of 17 GISTs of which 10 responded to imatinib (imatinib-sensitive) and seven progressed on imatinib (imatinib-resistant) [23]. They identified ten differentially expressed miRNAs a.o. miR-125a-5p that were found to be overexpressed in IM-r GIST and of which the expression was inversely correlated to levels of protein tyrosine phosphatase, non-receptor Type18 (PTPN18) [23]. The lowered PTPN18 levels conferred imatinib resistance in GIST822 cells. In a recent follow-up paper evidence was provided that the miR-125a-5p and PTPN18 effects on imatinib resistance were mediated through phosphorylated FAK levels [25]. Shi et al., reported downregulation of miR-518a-5p in IM-r that targets PIK3C2A [24]. Unfortunately, PIK3C2A levels were not modulated to validate its levels affecting imatinib sensitivity in GIST cells. Similarly, no evidence for an inverse correlation between miR-518a-5p and PIK3C2A expression in clinical samples was presented. The overlap in miRNAs detected between these studies and ours is limited, most likely due to different experimental set-ups, including the exact nature and number of GIST samples analyzed and the use of different miRNA detection platforms. In chronic myeloid leukemia (CML), another malignancy that is treated with imatinib, miRNAs have also been linked to imatinib resistance [26,27,28]. A number of miRNAs, e.g., miR-99a, miR-30c, and miR-101, which were all found downregulated in the IM-r samples (Table S6), have been previously associated with imatinib resistance in GIST or chronic myeloid leukemia [26,27]. The observed downregulation of miR-30c and miR-181a in our IM-r samples corresponded to findings in CML in which lowered expression of these miRNAs was also found in imatinib resistant cells [27,28]. In most cases dysregulated miRNAs in the IM-r setting were not further functionally characterized to substantiate their roles and involvement in imatinib resistance. 

Interestingly, the miRNA expression profiles were able to distinguish IM-r GIST samples with and without secondary KIT mutations. This observation may reflect a different biology underlying the resistance phenotype in the two groups. However, the accompanying fold differences in miRNA expression are small. To verify our findings larger sample cohorts should be examined using an RT-PCR platform.

Pathway and network analyses using differentially expressed transcripts and mRNAs as input indicated the upregulation of multiple cell-cycle related genes in the IM-r GIST samples. The cyclins A and E are well-known regulators of G1/S, S, and G2/M transition phases. Their increased expression levels, as well as those of most other genes in the network, most likely facilitates cell cycling and consequently cell proliferation. In this context the reduced expression of cyclin dependent kinase inhibitor 1 C (*CDKN1C*), a negative regulator of cell proliferation, also makes sense. However, the reduced expression of cyclin D2 (CCND2) does not seem to fit as its expression was found downregulated in the IM-r samples. It is unclear to what extent these findings are merely a reflection of the progressive nature of the IM-r GISTs. Aberrant expression of the majority of these genes is known to be involved in drug-resistance in various cancer types [29,30,31]. Of interest is the increased expression of the atypical E2F transcription factor family members E2F7 and E2F8. The precise function of these E2F family members in GIST and other cancers is still ill-defined. E2F7 overexpression has been linked to tamoxifen and anthracycline resistance in breast cancer and head and neck squamous cell carcinoma, respectively [32,33]. E2F8 promotes cell proliferation and tumorigenicity in breast cancer [34] and cisplatin resistance to estrogen receptor positive breast cancer cells [35]. The increased cell cycle activity may render IM-r GIST sensitive to cell cycle inhibitors.

The other highlighted gene interaction network is also conducive of cell cycle progression. This network points to central roles for AURKB and FOXM1 of which the expression was increased in IM-r GISTs. AURKB, together with AURKA, which is also upregulated in IM-r samples (Table S3), are serine/threonine kinases that regulate mitosis. These genes are found upregulated in many cancers and targeted inhibitors have been developed [36]. In GIST AURKA expression has been identified as a negative prognostic factor [37,38] and has recently been implicated as a therapeutic target [39]. The significance of FOXM1 in GIST was recently emphasized by reporting its role in GIST progression [40]. Furthermore the FOXO3a–FOXM1 axis has been implicated in cancer related processes like proliferation, survival, drug resistance, angiogenesis, migration, and DNA repair in other cancers [41]. Perhaps FOXM1 overexpression can be therapeutically exploited, e.g., by using thiazole antibiotics.

The integrative network analyses implicated some of the differentially expressed miRNAs as regulators of cell cycle related genes. Of special interest is miR-92a-3p, which is predicted to target *CDKN1C* through a highly conserved binding site in its 3’UTR, as predicted by TargetScan v7.2 (http://www.targetscan.org).The downregulation of miR-99a-5p affects mTOR levels [42,43]. The upregulation of mTOR stimulates cell cycle progression through its cell growth effectors S6K1 and eIF4E [44]. Finally, miR-101-3p has been implicated in imatinib sensitivity in CML with high levels sensitizing to imatinib through the downregulation of JAK2 and inhibition of NF-κB target genes [26]. So conversely miR-101-3p downregulation might cause imatinib resistance. Furthermore miR-101-3p regulates the PI3K/AKT/mTOR pathway [45,46] mediating AKT activation, which may reduce CDKN1C levels [47]. 

Our findings demonstrated that IM-r GIST samples can be distinguished from IM-n GIST samples based on their miRNA and mRNA expression profiles. In addition, we identified several miRNAs that discriminated between IM-r GIST samples with or without secondary KIT mutations. Pathway and network analyses highlighted cell cycle related genes/gene networks in IM-r GISTs and identified overexpressed proteins that may be pharmacologically targeted using small molecule inhibitors. Further, our data implicated at least three miRNAs, miR-92a-3p, miR-99a-5p, and miR-101-3p, as potential effectors of imatinib resistance. Future experimental in vitro and in vivo studies are needed to further substantiate and validate these findings.

## 4. Materials and Methods 

### 4.1. Patient Samples 

Fresh frozen GIST samples (n = 53) were obtained from the tissue bank of the Department of Pathology of the University Hospitals Leuven, Belgium and the Department of Soft Tissue/Bone Sarcoma and Melanoma, Marie Skłodowska-Curie Institute, Oncology Center, Warsaw, Poland. The initial GIST diagnosis was based on histological features as assessed by an expert pathologist, immunostaining for CD117/KIT and anoctamin (ANO1 or DOG1), and the presence of *KIT* mutations. All tumor samples that were analyzed contained >80% tumor cells, contained *KIT* activating mutations, and were derived from both IM-n (n = 33) and IM-r (n = 20) GISTs. The pathological and initial diagnostic molecular evaluation were all performed in a single institution (KU Leuven). Clinicopathological characteristics concerning patients and tumors are listed in Table 1. The majority of the patients from whom the GIST samples were derived were diagnosed and treated from 2000 to 2011 according to the applicable guidelines in that time-period. All patients consented to use their tissues for research purposes and approval was obtained from the Ethics Committee of the University Hospitals Leuven (ML7481) and the Oncology Center, Warsaw, Poland. The study was carried out in the context of a research protocol “Translational research in soft tissue sarcomas”, which was reviewed and approved by the Medical Ethical Review board of the Erasmus Medical Center (MEC-2016-213) on 11th April 2016. The study was performed in accordance with the Declaration of Helsinki.

### 4.2. RNA Isolation and microRNA Profiling

Total RNA was isolated from fresh frozen tumor samples using RNAbee (Tel Test Inc., Friendswood, TX, USA) following the standard extraction protocol recommended by the manufacturer. RNA concentration and quality were examined using a Nanodrop-1000 (Nanodrop Technologies, Wilmington, DE, USA). MiRNA expression profiles were determined using miRNA microarrays, essentially as described before by Pothof et al. [48]. In brief, using the Kreatech ULS^TM^ aRNA labeling Kit (Kreatech Diagnostics/Leica Biosystems, Amsterdam, the Netherlands), 1 μg total RNA was labeled with Cy3. The Cy3-tagged RNA was hybridized overnight to LNA™ modified oligonucleotide capture probes (Exiqon, Vedbaek, Denmark) spotted in duplicate on Nexterion E slides. Of the 1344 capture probes on the slides, 725 were specifically designed to detect human miRNAs. After hybridization, slides were scanned, and median spot intensity was determined using ImaGene software (BioDiscovery Inc., El Segundo, CA, USA). After background subtraction, expression values were Quantile normalized using R-software, bad spots were deleted, and duplicate spots averaged. The normalized miRNA expression data were log 2 transformed and median centered to acquire the relative expression values that were used for hierarchical clustering analysis using the open source software Cluster 3.0 [49] and Java Tree View [50]. A two-sample t-test was used to determine statistical significance (*p* < 0.05) between imatinib-naïve and imatinib-resistant samples and the Benjamini–Hochberg false discovery rate (FDR) was used to control for multiple testing. The miRNA expression datasets generated and analyzed during the current study are presented in Table S7.

### 4.3. mRNA Expression Analysis

Gene expression analysis using the Affymetrix HG-U133_Plus_2 platform was carried out according to standard operating procedures by the VIB MicroArray Facility of the KU Leuven. Raw. cel files were processed using fRMA parameters (median polish) after which batch effects were corrected using ComBat. [51,52]. BRB-Array tools (Biometric Research Branch Array Tools (http://brb.nci.nih.gov/BRB-ArrayTools/) was used for analyzing the transcript expression data and a two-sample t-test was used for statistical testing. The mRNA expression datasets generated and analyzed during the current study have been deposited to the Gene Expression Omnibus (GEO) data repository under accession number GSE132542.

### 4.4. Quantitative RT-PCR

The differential expression of selected miRNAs in IM-n (n = 33) and IM-r (n = 20) GIST samples, as detected by the LNA™ modified oligonucleotide platform, was validated by RT-PCR using the TaqMan^®^ miRNA Assays Technology (Applied Biosystems/ThermoFisher Scientific, Bleiswijk, the Netherlands). In brief, total RNA (50 ng) was reverse transcribed in a multiplex reaction using specific miRNA primers from the TaqMan^®^ miRNA Assays and reagents from the TaqMan^®^ miRNA Reverse Transcription Kit (Applied Biosystems/ThermoFisher Scientific) according to the manufacturer’s protocol. The resulting cDNA was used as input in a quantitative real-time PCR (qPCR) using a miRNA specific primer/probe mix together with the TaqMan^®^ Universal PCR Master Mix No AmpErase^®^ UNG (Applied Biosystems/ThermoFisher Scientific) using the 7500 Fast Real-Time PCR systems (Applied Biosystems). The qPCR data were analyzed using SDS software (version 2.4, Applied Biosystems/ThermoFisher Scientific). A standard dilution series of a cDNA sample-pool was included on every plate allowing for the absolute quantification of the miRNA expression. 

The differential expression of selected mRNAs in IM-n (n = 33) and IM-r (n = 20) GIST samples, as detected by the Affymetrix platform, was validated by RT-PCR using the TaqMan^®^ Technology (Applied Biosystems/ThermoFisher Scientific). In brief, total RNA (1 µg) was used as input for a reverse transcription reaction using a high capacity cDNA reverse transcription kit (Applied Biosystems/ThermoFisher Scientific) according to procedures by the manufacturer. The cDNA was used in a PCR reaction using primer/probe combinations from the following Taqman^®^ gene expression assays (AURKA, assay ID: Hs01582072_m1; AURKB, assay ID: Hs00945858_g1; CCND2, assay ID:Hs00153380_m1; CCNE2, assay ID: Hs00180319_m1; CDK1, assay ID: Hs00938777_m1; CDKN1C, assay ID: Hs00175938_m1; E2F7, assay ID: Hs00987777_m1; FOXM1, assay ID: Hs01073586_m1) and Taqman^®^ Universal PCR master mix using the 7500 Fast Real-Time PCR system (all obtained from Applied Biosystems/ThermoFisher Scientific) according to the manufacturer’s recommendations. Three housekeepers (GAPDH, HPRT, and PPIA) were used for normalization purposes using the comparative C_T_-method. The qPCR data were analyzed using SDS software (version 2.4, Applied Biosystems/ThermoFisher Scientific). 

### 4.5. Pathway Analysis

For pathway analyses, a commercial software application, Ingenuity^®^ Pathway Analysis (IPA^®^), was used. IPA calculates and visualizes the known pathway associations and interactions between sets of transcripts. mRNAs and/or miRNAs that were significantly differentially expressed between IM-n and IM-r samples were selected and accompanying identifiers and fold changes were uploaded into the IPA. The mRNA data were used to identify canonical signaling and metabolic pathways that were predicted to be activated or inhibited (canonical pathway analysis). The miRNA and mRNA data together were used to construct interaction networks, networks based on molecular relationships between differentially expressed mRNAs and/or miRNAs. These networks were matched to and derived from a “global molecular network” developed from the available online information in the IPA. The pathway and network analyses were performed using filtering of “Human” and “uncategorized” for species as well as “direct and indirect relationships” for general settings. The presented networks were representations of molecular relationships between mRNA–mRNA and miRNA–mRNA interactions. 

## Figures and Tables

**Table 1:** Patient and tumor characteristics.

**Gastrointestinal Stromal Tumors Imatinib-Naïve (IM-n)**						
**Male**	n = 23					
**Female**	n = 10					
**Median age (range)**	65 (41–85)					
Sample code	**KIT mutation status**		**Location**	Risk of malignancy *	**miRNA**	**mRNA**
**X1KIT11**	p.V560D/KIT11		Small intestine	intermediate	✓	✓
**X4KIT11**	p.W557_V559delinsF/KIT11		Small intestine	high	✓	✓
**X6KIT11**	p.W557R/KIT11		Small intestine	overtly malignant **	✓	
**X8KIT11**	p.L576_R588dup/KIT11		Stomach	intermediate	✓	✓
**X9.2.KIT11**	p.W557_V559delinsF/KIT11		Stomach	high	✓	
**X10KIT11**	p.W557R/KIT11		Stomach	intermediate	✓	
**X12KIT11**	p.K550_V555del/KIT11		Stomach	high	✓	
**X14KIT11**	p.581_590insKWEFPRNRLS/KIT11		Stomach	intermediate	✓	✓
**X23KIT11**	p.W557_K558del/KIT11		Stomach	intermediate	✓	
**X24KIT11**	p.V554D/KIT11		Stomach	intermediate	✓	✓
**X25KIT11**	p.W557_G592dup (c.1669_1774 + 2dup)/KIT11		Stomach	high	✓	
**X26KIT11**	p.K558_V559delinsN (AAT) homo/KIT11		Mediastinum	high	✓	
**X34KIT11**	p.W557_V560delinsF/KIT11		Stomach	high	✓	
**X35KIT11**	p.V560D/KIT11		Stomach	overtly malignant	✓	✓
**X39KIT11**	p.L576P/KIT11		Duodenum	intermediate	✓	✓
**X40KIT9**	p.A502_Y503dup/KIT9		Colon	overtly malignant	✓	
**X45KIT11**	p.K550_K558delinsG/KIT11		Small intestine	overtly malignant	✓	
**X47KIT11**	p.V559A/KIT11		Stomach	low	✓	
**X48KIT11**	p.V560A/KIT11		Duodenum	intermediate	✓	✓
**X53KIT11**	p.Q556_V559delinsH; c.1668_1676del9/KIT11		Stomach	overtly malignant	✓	✓
**X55KIT11**	p.W557_K558del/KIT11		Stomach	intermediate	✓	
**X64KIT11**	p.V560D/KIT11		Stomach	high	✓	✓
**X78KIT11**	p.W557_K558del; c.1669_1674del/KIT11		Stomach	overtly malignant	✓	✓
**X82KIT11**	p.W557_P573delinsFQ/KIT11		Stomach	overtly malignant	✓	
**X86KIT9**	p.A502_Y503dup/KIT9		Small intestine	overtly malignant	✓	
**X89KIT17**	p.N822K/KIT17		Small intestine	overtly malignant	✓	✓
**X95KIT11**	p.T574_R586insK/KIT11		Stomach	intermediate	✓	
**X100KIT9**	p.A502_Y503dup/KIT9		Small intestine	overtly malignant	✓	
**X101KIT11**	p.E554_K558del/KIT11		Stomach	low	✓	
**X102KIT11**	p.W557R/KIT11		Stomach	high	✓	✓
**X108KIT9**	p.A502_Y503dup/KIT9		Small intestine	overtly malignant	✓	✓
**X.KIT11**	p.M552_E554delinsK/KIT11		Small intestine	overtly malignant	✓	
**X119KIT11**	p.Q556_I563del/KIT11		Stomach	low	✓	
Gastrointestinal Stromal Tumors Imatinib-Resistant (IM-r) ***						
**Male**	n = 14					
**Female**	n = 6					
**Median age (range)**	49.5 (22–67)					
Sample code	**KIT mutation status**	**KIT secondary mutation**	**Location**	Risk of malignancy *	**miRNA**	**mRNA**
**X2KIT9**	p.A502_Y503dup/KIT9	Not detected	Colon	overtly malignant	✓	✓
**X27KIT11**	p.L576P; c.1727 T > C/27KIT11	p.D820Y; c.2458G > T	Small intestine	overtly malignant	✓	✓
**X36KIT11**	p.Q556_E561delinsQ/KIT11	Not detected	Small intestine	overtly malignant	✓	✓
**X42KIT9**	p.A502_Y503dup/KIT9	KIT: p.V654A	Small intestine	overtly malignant	✓	✓
**X44KIT9**	p.A502_Y503dup/KIT9	Not detected	Small intestine	overtly malignant	✓	✓
**X54KIT11**	p.K550_K558delinsQ/KIT11	KIT: p.D820Y	Small intestine	overtly malignant	✓	✓
**X58KIT11**	p.I563_Q575del/KIT11	KIT: p.D820Y	Stomach	overtly malignant	✓	✓
**X70KIT11**	p.E554_D572del/KIT11	KIT: p.V654A	Small intestine	overtly malignant	✓	✓
**X71KIT11**	p.V559D/KIT11	KIT: p.D820G	Small intestine	overtly malignant	✓	✓
**X73KIT11**	p.N567_L576delinsI/KIT11	Not detected	Small intestine	overtly malignant	✓	✓
**X74KIT9**	p.A502_Y503dup/KIT9	Not detected	Small intestine	overtly malignant	✓	
**X77KIT9**	p.A502_Y503dup/KIT9	Not detected	Small intestine	overtly malignant	✓	✓
**X85KIT9**	p.A502_Y503dup/KIT9	Not detected	Small intestine	overtly malignant	✓	✓
**X91KIT11**	p.K550_K558del/KIT11	KIT: p.D820Y	Small intestine	overtly malignant	✓	
**X104KIT11**	p.W557_K558del/KIT11	Not detected	Small intestine	overtly malignant	✓	✓
**X105KIT9**	p.A502_Y503dup/KIT9	Not detected	Small intestine	overtly malignant	✓	✓
**X112KIT11**	c.1654_1671del18 (p.M552_W557del)/KIT11	Not detected	Small intestine	overtly malignant	✓	✓
**X116KIT11**	p.557_558del homo/KIT11	Not detected	Small intestine	overtly malignant	✓	
**X117KIT11**	p.K550_V555delinsL; c.1648_1663delinsT/KIT11	p.D820Y; c.2458 G > T	Small intestine	overtly malignant	✓	
**X118KIT11**	p.V559D; c.1676 T > A/KIT11	KIT p.V654A; c.1961 T > C	Small intestine	overtly malignant	✓	

* Tumor risk assessment was performed using AFiP criteria (Miettinen M. & Lasota, J. *Semin. Diagn. Pathol*. **2006**, 23, 70–83); ** Recurrent or metastatic disease during clinical follow-up; *** Patients were only treated with imatinib, progression occurred after 6 months.

## Supplementary Materials

The following are available online at https://www.mdpi.com/2072-6694/11/6/882/s1: Table S1: Differentially expressed microRNAs between imatinib-naïve and imatinib-resistant gastrointestinal stromal tumors; Table S2: Differentially expressed microRNAs between imatinib-resistant gastrointestinal stromal tumors with and without secondary KIT mutations; Table S3: List of 352 differentially expressed genes between imatinib-resistant and imatinib-naïve GIST samples; Table S4: Differentially expressed genes associated with the top deregulated canonical pathways; Table S5: Ingenuity Pathway Analysis Symbols; Table S6: Differentially expressed microRNAs between imatinib-naïve and imatinib-resistant gastrointestinal stromal tumors in the samples that were used for mRNA profiling; Table S7: Overview of the microRNA expression levels measured in the imatinib-naïve and imatinib-resistant gastrointestinal stromal tumor samples; Figure S1: Differentially expressed mRNAs between imatinib-naïve and imatinib-resistant GIST samples; Figure S2: Top deregulated canonical pathways between imatinib-naïve and imatinib-resistant gastrointestinal stromal tumors; Figure S3: Quantitative RT-PCR validation of differentially expressed microRNAs and mRNAs in imatinib-naïve and imatinib-resistant gastrointestinal stromal tumors.
