# Supplementary material for: Molecular Comparison of Imatinib-Naïve and Resistant Gastrointestinal Stromal Tumors: Differentially Expressed microRNAs and mRNAs
Source: Cancers (Basel). 2019 Jun 24;11(6):882. doi: 10.3390/cancers11060882 (PMC6627192; doi:10.3390/cancers11060882)
Supplement: Supplementary file 1 [file cancers-11-00882-s001.zip › cancers-520397-Supplementary/Legends and Tables S1,S2,S4,S6.pdf]

# Supplementary Information

**Table S1.** Differentially expressed microRNAs between imatinib-naïve and imatinib-resistant gastrointestinal stromal tumors. Listed are 35 miRNAs ($p < 0.01$; FDR < 20%) that are differentially expressed between imatinib-naïve (IM-n) ($n = 33$) and imatinib-resistant (IM-r) GIST ($n = 20$) samples and that were used in the supervised hierarchical clustering (Figure 1). *p*-values of *t*-test (parametric P) and false discovery rate *p*-values (FDR) are listed, as well as the fold-change upregulation of miRNA expression (Up in IM-r and Up in IM-n).

| | miRNA | Up in IM-r | Up in IM-n | Parametric P | FDR |
|---|---|---|---|---|---|
| **1** | miR-186 | | 1.3495141 | $5.98286 \times 10^{-5}$ | 0.038568 |
| **2** | miR-526a;miR-520c-5p; miR-518d-5p | 1.2326554 | | 0.000106836 | 0.038568 |
| **3** | miR-188-5p | 2.0378778 | | 0.000345567 | 0.083167 |
| **4** | miR-675 | | 1.6985022 | 0.000863845 | 0.15053 |
| **5** | miR-1296 | | 1.2730468 | 0.001263595 | 0.15053 |
| **6** | miR-708* | 1.2024122 | | 0.001281441 | 0.15053 |
| **7** | miR-24 | | 1.4884172 | 0.001632288 | 0.15053 |
| **8** | miR-455-3p | | 1.5224393 | 0.002012757 | 0.15053 |
| **9** | miR-335 | 1.9508284 | | 0.002150029 | 0.15053 |
| **10** | miR-92a | 1.3374119 | | 0.002282718 | 0.15053 |
| **11** | miR-18b | | 1.3447477 | 0.002293387 | 0.15053 |
| **12** | miR-144 | 1.6089693 | | 0.00322487 | 0.173452 |
| **13** | miR-629 | 1.2127094 | | 0.003348348 | 0.173452 |
| **14** | miR-376b | | 1.3186401 | 0.003363339 | 0.173452 |
| **15** | miR-885-5p | 1.3111764 | | 0.003899124 | 0.18163 |
| **16** | miR-143* | | 1.4948171 | 0.004266206 | 0.18163 |
| **17** | miR-891a | 1.2480536 | | 0.00428809 | 0.18163 |
| **18** | miR-662 | 1.1984868 | | 0.004690577 | 0.18163 |
| **19** | miR-539 | | 1.2473837 | 0.004810446 | 0.18163 |
| **20** | miR-212 | 1.3433722 | | 0.005607983 | 0.18163 |
| **21** | miR-30c | | 1.3199678 | 0.005845988 | 0.18163 |
| **22** | miR-18a* | 1.185796 | | 0.005863012 | 0.18163 |
| **23** | miR-505* | 1.2471201 | | 0.006096226 | 0.18163 |
| **24** | miR-542-5p | 1.201965 | | 0.006223562 | 0.18163 |
| **25** | miR-148b | | 1.2824882 | 0.006527379 | 0.18163 |
| **26** | miR-181a | | 1.3637022 | 0.006540709 | 0.18163 |
| **27** | miR-516a-3p;miR-516b* | 1.1586478 | | 0.007321078 | 0.190193 |
| **28** | miR-99a | | 1.5850102 | 0.00762179 | 0.190193 |
| **29** | miR-548b-5p | | 1.1389362 | 0.007639334 | 0.190193 |
| **30** | miR-23b | | 1.3400315 | 0.008718018 | 0.199662 |
| **31** | miR-452 | | 1.3033082 | 0.008822402 | 0.199662 |
| **32** | miR-641 | 1.1238858 | | 0.009550755 | 0.199662 |
| **33** | miR-144* | 1.1791067 | | 0.009642671 | 0.199662 |
| **34** | miR-195* | 1.3858141 | | 0.009665876 | 0.199662 |
| **35** | miR-595 | 1.3852625 | | 0.009678903 | 0.199662 |

**Table S2.** Differentially expressed microRNAs between imatinib-resistant gastrointestinal stromal tumors with and without secondary KIT mutations. Listed are 22 miRNAs ($p < 0.01$) that distinguish between IM-r GIST samples ($n = 9$) with resistance causing secondary KIT mutations ($S^+$) and IM-r GIST ($n = 11$) samples without secondary mutations ($S^-$) and that were used in a supervised hierarchical clustering (Figure 2). *p*-values of *t*-test (Parametric P) and false discovery rate p-values (FDR) are listed, as well as the fold-change upregulation of miRNA expression (Up in $S^+$ and Up in $S^-$).

| | **miRNA** | **Up in $S^-$** | **Up in $S^+$** | **Parametric P** | **FDR** |
|---|---|---|---|---|---|
| **1** | miR-550* | 1.499389 | | 0.000429914 | 0.310398 |
| **2** | miR-618 | | 1.274545 | 0.00102463 | 0.349131 |
| **3** | miR-196b | | 1.393345 | 0.001450684 | 0.349131 |
| **4** | miR-181b | | 1.44845 | 0.003302656 | 0.441227 |
| **5** | miR-578 | | 1.396228 | 0.003455634 | 0.441227 |
| **6** | miR-302d | | 1.146457 | 0.003666707 | 0.441227 |
| **7** | miR-380* | | 1.432246 | 0.004278633 | 0.44131 |
| **8** | miR-581 | | 1.316223 | 0.005066014 | 0.457208 |
| **9** | miR-621 | 1.120689 | | 0.009139269 | 0.481995 |
| **10** | miR-32 | | 1.199577 | 0.009151744 | 0.481995 |
| **11** | miR-566 | 1.173189 | | 0.009257969 | 0.481995 |
| **12** | miR-26b* | | 1.124953 | 0.009916984 | 0.481995 |
| **13** | miR-519c-3p | | 1.277479 | 0.009945044 | 0.481995 |
| **14** | miR-301b | | 1.18747 | 0.010807049 | 0.481995 |
| **15** | miR-640 | 1.239771 | | 0.010978027 | 0.481995 |
| **16** | miR-496 | | 1.204576 | 0.01099503 | 0.481995 |
| **17** | miR-125b | | 1.824483 | 0.011348924 | 0.481995 |
| **18** | miR-335* | | 1.30212 | 0.01270197 | 0.50949 |
| **19** | miR-191* | | 1.231575 | 0.014321017 | 0.522105 |
| **20** | miR-515-5p | 1.33432 | | 0.014630895 | 0.522105 |
| **21** | miR-652 | 2.117462 | | 0.015185871 | 0.522105 |
| **22** | miR-609 | | 1.259427 | 0.016334917 | 0.531633 |

**Table S3.** List of 352 differentially expressed genes between imatinib-resistant and imatinib-naïve GIST samples. Comparison of mRNA expression data from IM-r ($n = 15$) and IM-n ($n = 14$) GIST samples identified 352 genes, defined by 475 different Affymetrix probe sets, that were differentially expressed ($p < 0.008$; FDR < 10%) (Supplementary Figure 1). Listed for each gene in the table are the Affymetrix probes set identifiers (NAME), fold-differences in expression (IM-r vs IM-n), p-values (Parametric P), False Discovery Rates (FDR), gene ID (Representation) and gene symbols.

**Table S4.** Differentially expressed genes associated with the top deregulated canonical pathways. Among 352 most differentially expressed genes, 8 genes were among the most differentially expressed ones in the top deregulated canonical pathways associated with the cell cycle (Supplementary Figure 2). Listed are gene symbols and the corresponding Entrez gene names. The *p* values—based on two-sample *t*-test—indicate the statistical significance of the genes between IM-r and IM-n GIST samples. The depicted fold changes (FC) were calculated from the comparison of the Geomean of the gene expression in IM-r and IM-n groups. Minus and plus values signify the lower and higher expression of the corresponding genes in the IM-r setting, respectively.

| **Symbol** | **Entrez Gene Name** | ***p*-value** | **FC IM-r vs IM-n** |
|---|---|---|---|
| **CCNA2** | cyclin A2 | 0.000615 | 2.632 |
| **CCNB1** | cyclin B1 | 0.0066 | 2.326 |
| **CCNB2** | cyclin B2 | 0.00811 | 2.128 |
| **CCND2** | cyclin D2 | 0.000645 | -5.36 |
| **CCNE2** | cyclin E2 | 0.0000975 | 2.941 |
| **CDK1** | cyclin dependent kinase 1 | 0.00322 | 3.03 |
| **E2F7** | E2F transcription factor 7 | 0.00216 | 2.174 |
| **E2F8** | E2F transcription factor 8 | 0.00102 | 2.041 |

**Table S5.** Ingenuity Pathway Analysis Symbols.

**Table S6.** Differentially expressed microRNAs between imatinib-naïve and imatinib-resistant gastrointestinal stromal tumors in the samples that were used for mRNA profiling. Eighty-eight significantly ($p < 0.03$; FDR < 30%) differentially expressed miRNAs were detected in 29 GIST samples (IM-r, $n = 15$ vs IM-n, $n = 14$) that were also used for mRNA profiling. The 88 differentially expressed miRNAs were used as input for the integrative IPA of Figure 5. *p*-values of *t*-test (Parametric P) and false discovery rate p-values (FDR) are listed, as well as the fold-change upregulation of miRNA expression (Up in IM-r and Up in IM-n).

| | miRNA | Up in IM-r | Up in IM-n | Parametric P | FDR |
|---|---|---|---|---|---|
| **1** | miR-186 | | 1.465364413 | 0.000142123 | 0.065297868 |
| **2** | miR-23b | | 1.600990618 | 0.000315439 | 0.065297868 |
| **3** | miR-30c | | 1.569388509 | 0.000406921 | 0.065297868 |
| **4** | miR-374a | | 1.399173478 | 0.000481105 | 0.065297868 |
| **5** | miR-1296 | | 1.36939107 | 0.000587714 | 0.065297868 |
| **6** | miR-99a | | 2.295847867 | 0.000652128 | 0.065297868 |
| **7** | miR-30e | | 1.360999043 | 0.000755959 | 0.065297868 |
| **8** | miR-708* | 1.220111545 | | 0.000836728 | 0.065297868 |
| **9** | miR-24 | | 1.70013927 | 0.000882683 | 0.065297868 |
| **10** | miR-374b | | 1.238498341 | 0.000903152 | 0.065297868 |
| **11** | miR-662 | 1.263511315 | | 0.000999104 | 0.065668409 |
| **12** | miR-338-3p | 1.290656116 | | 0.001370818 | 0.077562569 |
| **13** | miR-511 | | 1.132611196 | 0.001394624 | 0.077562569 |
| **14** | miR-629 | 1.328014266 | | 0.001785574 | 0.092212146 |
| **15** | miR-192* | | 1.185132714 | 0.002217836 | 0.096316515 |
| **16** | miR-34c-5p | 1.304273962 | | 0.002251631 | 0.096316515 |
| **17** | miR-455-5p | | 1.591962841 | 0.002264704 | 0.096316515 |
| **18** | miR-891a | 1.304766558 | | 0.002702779 | 0.108561636 |
| **19** | miR-524-3p | | 1.340586356 | 0.003121461 | 0.114789794 |
| **20** | miR-125a-5p | | 1.471858759 | 0.003389134 | 0.114789794 |
| **21** | miR-526a; miR-520c-5p; miR-518d-5p | 1.213370815 | | 0.003425339 | 0.114789794 |
| **22** | miR-425* | 1.313142787 | | 0.003492912 | 0.114789794 |
| **23** | miR-661 | 1.213995299 | | 0.003922538 | 0.120973061 |
| **24** | miR-675 | | 1.661244547 | 0.004015703 | 0.120973061 |
| **25** | miR-885-5p | 1.399970729 | | 0.004224052 | 0.122159595 |
| **26** | miR-455-3p | | 1.66339195 | 0.004585885 | 0.127522877 |
| **26** | miR-493* | 1.39876134 | | 0.007619979 | 0.203048448 |
| **28** | miR-635 | 1.232138082 | | 0.007863564 | 0.203048448 |
| **29** | miR-30a | | 1.588884872 | 0.008520114 | 0.212415261 |
| **30** | miR-202* | | 1.284870076 | 0.0094041 | 0.218726806 |
| **31** | miR-501-3p | | 1.447609026 | 0.009424178 | 0.218726806 |
| **32** | miR-106a* | 1.410743402 | | 0.009904977 | 0.218726806 |
| **33** | miR-595 | 1.622629452 | | 0.010108567 | 0.218726806 |
| **34** | miR-204 | | 1.145140088 | 0.010576345 | 0.218726806 |
| **35** | miR-132* | 1.252451393 | | 0.010788021 | 0.218726806 |
| **36** | miR-29c | | 1.435810249 | 0.011432348 | 0.218726806 |
| **37** | miR-181a | | 1.387856624 | 0.011456468 | 0.218726806 |
| **38** | miR-641 | 1.128133915 | | 0.011496015 | 0.218726806 |
| **39** | miR-525-5p | 1.310391793 | | 0.012919296 | 0.230343614 |
| **40** | miR-524-5p | 1.237620101 | | 0.013002086 | 0.230343614 |
| **41** | miR-584 | | 1.22054574 | 0.013062363 | 0.230343614 |
| **42** | miR-190 | | 1.309696336 | 0.014025091 | 0.241431918 |
| **43** | miR-220c | 1.287503569 | | 0.015363205 | 0.249776498 |
| **44** | miR-135b* | 1.431042466 | | 0.015727909 | 0.249776498 |
| **45** | miR-144 | 1.56789822 | | 0.015855044 | 0.249776498 |
| **46** | miR-330-5p | 1.333775505 | | 0.015891727 | 0.249776498 |
| **47** | miR-136 | 1.351326777 | | 0.016292595 | 0.250628642 |
| **48** | miR-657 | 1.311175418 | | 0.017027307 | 0.253034294 |
| **49** | miR-520c-3p | 1.371534698 | | 0.017148936 | 0.253034294 |

| | | | | | |
|---|---|---|---|---|---|
| **50** | miR-625* | | 1.803149424 | 0.017805656 | 0.257469782 |
| **51** | let-7f | | 1.753102735 | 0.018310513 | 0.259578453 |
| **52** | miR-30c-1* | | 1.28614592 | 0.019764184 | 0.27479818 |
| **53** | miR-144* | 1.194803304 | | 0.020216862 | 0.275788508 |
| **54** | miR-541 | 1.198409739 | | 0.020768723 | 0.278070128 |
| **55** | miR-520a-5p | 1.228522565 | | 0.02186297 | 0.279390831 |
| **56** | miR-518f* | 1.194516641 | | 0.022728614 | 0.279390831 |
| **57** | miR-491-3p | | 1.655054252 | 0.022914672 | 0.279390831 |
| **58** | miR-26b | | 1.53087878 | 0.023108316 | 0.279390831 |
| **59** | miR-146b-3p | 1.282473253 | | 0.023670193 | 0.279390831 |
| **60** | miR-577 | | 1.200769137 | 0.024221263 | 0.279390831 |
| **61** | miR-155 | 1.375105134 | | 0.024332148 | 0.279390831 |
| **62** | miR-101 | | 1.441474426 | 0.024625483 | 0.279390831 |
| **63** | miR-516a-3p; miR516b* | 1.2039334 | | 0.025414319 | 0.279390831 |
| **64** | miR-30b | | 1.367010087 | 0.025901015 | 0.279390831 |
| **65** | miR-582-3p | 1.157495228 | | 0.02623892 | 0.279390831 |
| **66** | miR-431* | | 1.218489135 | 0.026409191 | 0.279390831 |
| **67** | miR-659 | 1.203371358 | | 0.026979188 | 0.279390831 |
| **68** | miR-885-3p | 1.376404732 | | 0.027352065 | 0.279390831 |
| **69** | miR-337-3p | | 1.135486605 | 0.027634654 | 0.279390831 |
| **70** | miR-29b | | 1.388370529 | 0.027731949 | 0.279390831 |
| **71** | miR-92a | 1.305186792 | | 0.027804244 | 0.279390831 |
| **72** | miR-890 | | 1.25279541 | 0.02894404 | 0.279390831 |
| **73** | miR-184 | 1.363654376 | | 0.029674786 | 0.279390831 |
| **74** | miR-182* | | 1.11840109 | 0.029871354 | 0.279390831 |
| **75** | miR-96* | 1.13454567 | | 0.030353262 | 0.279390831 |
| **76** | miR-505* | 1.325496038 | | 0.030411543 | 0.279390831 |
| **77** | miR-18b | | 1.32288215 | 0.03049196 | 0.279390831 |
| **78** | miR-220b | 1.270376441 | | 0.03112976 | 0.279390831 |
| **79** | miR-548b-5p | | 1.239066827 | 0.031162015 | 0.279390831 |
| **80** | miR-510 | 1.273879596 | | 0.031657895 | 0.279390831 |
| **81** | miR-379* | | 1.139422237 | 0.031668059 | 0.279390831 |
| **82** | miR-26a-2* | | 1.152867809 | 0.032021038 | 0.279390831 |
| **83** | miR-155* | | 1.328898801 | 0.032936027 | 0.279390831 |
| **84** | miR-18a* | 1.158960147 | | 0.033168697 | 0.279390831 |
| **85** | miR-150 | | 1.35609573 | 0.033285129 | 0.279390831 |
| **86** | miR-920 | 1.41268658 | | 0.033486091 | 0.279390831 |
| **87** | miR-616 | | 1.11727855 | 0.033619644 | 0.279390831 |
| **88** | miR-877* | 1.342330426 | | 0.035692417 | 0.293245652 |

**Table S7.** Overview of the microRNA expression levels measured in the imatinib-naïve and imatinib-resistant gastrointestinal stromal tumor samples. The colors in the top row indicate the IM-n samples (blue) and the IM-r samples (brown). The ID numbers in first column refer to specific miRNA capture probes as indicated in the second column. The ID annotation can also be found in the platform description in the Gene Expression Omnibus (GEO) data repository under accession number GPL16851. The sample designation corresponds to the annotation used throughout the manuscript. The values depicted are normalised miRNA expression values that were used for the analyses described in the manuscript.

**Figure S1.** Differentially expressed mRNAs between imatinib-naïve and imatinib-resistant GIST samples. Depicted is a heat map of a supervised hierarchical clustering based on the 475 Affymetrix probes sets that represent 352 most significant ($p < 0.008$; FDR < 10%) differentially expressed genes in fresh frozen GIST samples from IM-n ($n = 14$) and IM-r ($n = 15$) patients. In the heat map red indicates relative high expression and green indicates relative low expression.

**Figure S2.** Top deregulated canonical pathways between imatinib-naïve and imatinib-resistant gastrointestinal stromal tumors. The most deregulated canonical pathways in the IM-r samples were derived, using Ingenuity Pathway Analyses, from the 475 most differentially expressed mRNAs ($p < 0.008$; FDR < 10%) between IM-n and IM-r GIST samples. The significance of the top deregulated pathways, which is displayed on the top X-axis as ''-log(p-value)'' is an indication of the probability of the association of genes represented in our mRNA dataset with the canonical pathways by random chance alone. The orange colored bars are indicative of

predicted pathway activation (positive Z-score) and the blue ones indicate predicted inhibition (negative Z-score). White and gray bars are those with Z-score close to 0 or no available prediction, respectively. The ratio, indicated on the bottom X-axis, represents the number of genes in a given pathway that pass the cut-off criteria/number of total genes that constitute the pathway, and is visualized by orange points.

**Figure S3.** Quantitative RT-PCR validation of differentially expressed microRNAs and mRNAs in imatinib-naïve and imatinib-resistant gastrointestinal stromal tumors. **A**) A standardized RT-PCR procedure was used to validate the expression levels of differentially expressed microRNAs in the IM-n ($n$ = 33) and IM-r GIST ($n$ = 20) samples sets. A standard dilution series of a cDNA sample-pool was used to determine absolute quantification of the miRNA expression. **B**) A standardized RT-PCR procedure was used to validate the expression levels of differentially expressed mRNAs in the IM-n ($n$ = 33) and IM-r ($n$ = 20) GIST samples. Expression levels were analyzed using GAPDH, HPRT and PPIA for normalization purposes. Boxplots depict 1$^{st}$ and 3$^{rd}$ quartile with the median indicated as a horizontal line. Outliers designate measurements > 1.5 IQR (interquartile range). * indicates $p < 0.05$.
