# Supplementary material for: Molecular Comparison of Imatinib-Naïve and Resistant Gastrointestinal Stromal Tumors: Differentially Expressed microRNAs and mRNAs
Source: Cancers (Basel). 2019 Jun 24;11(6):882. doi: 10.3390/cancers11060882 (PMC6627192; doi:10.3390/cancers11060882)

Supplementary Table 5

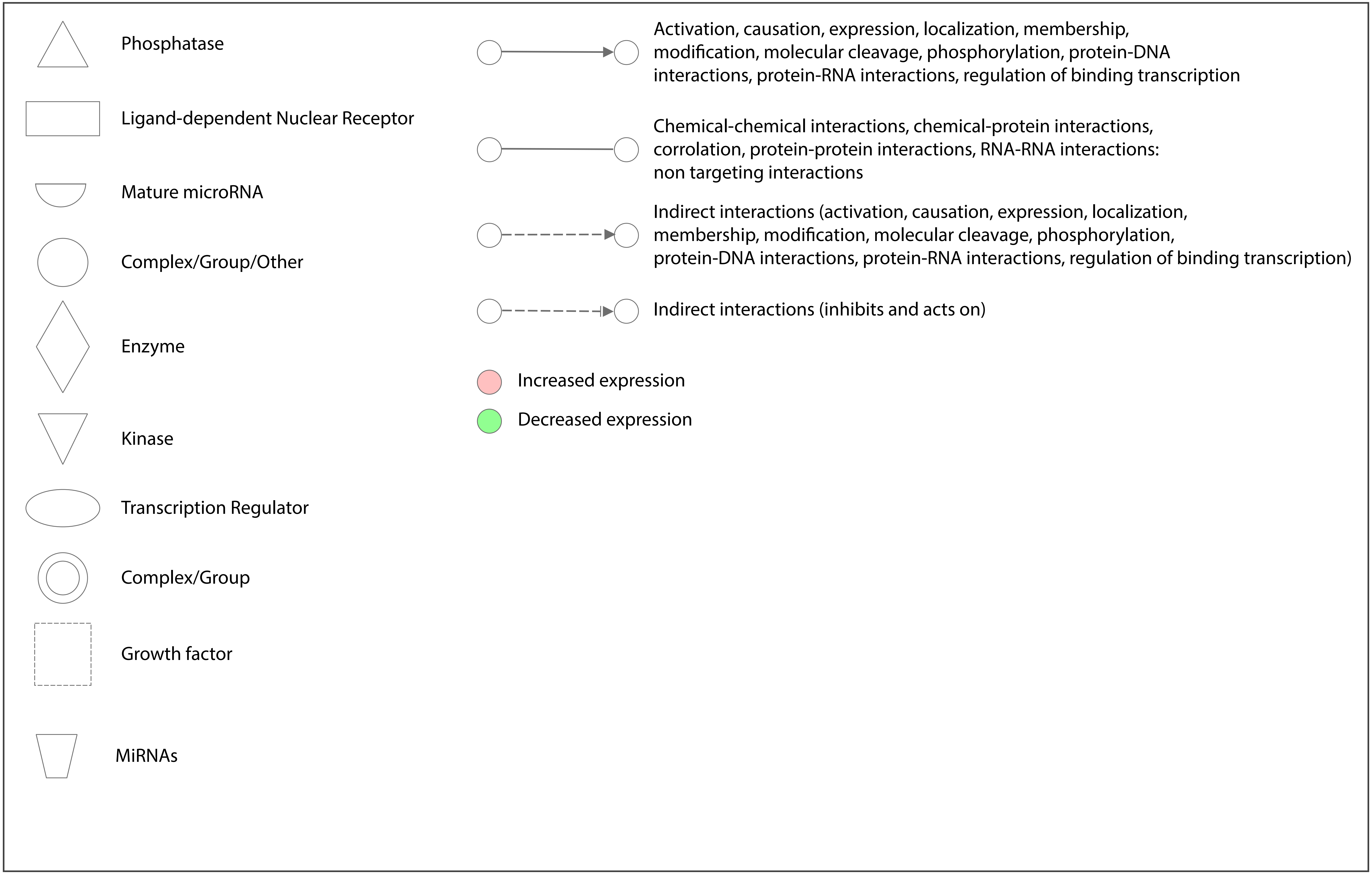

Supplement: Supplementary file 1 [file cancers-11-00882-s001.zip › cancers-520397-Supplementary/Table S5.pdf]
