# Supplementary figures and images for: Molecular Comparison of Imatinib-Naïve and Resistant Gastrointestinal Stromal Tumors: Differentially Expressed microRNAs and mRNAs
Source: Cancers (Basel). 2019 Jun 24;11(6):882. doi: 10.3390/cancers11060882 (PMC6627192; doi:10.3390/cancers11060882)

Supplementary Figure 1

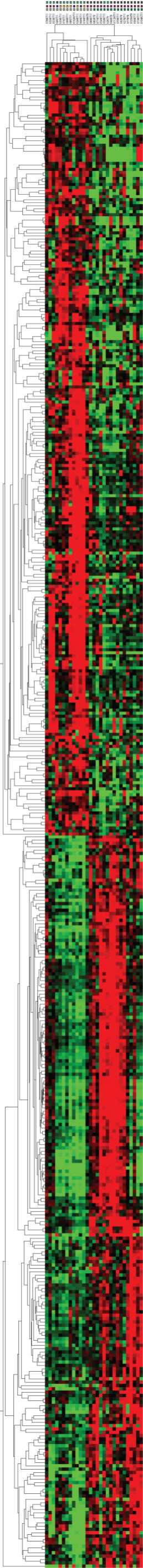

Supplement: Supplementary file 1 [file cancers-11-00882-s001.zip › cancers-520397-Supplementary/Figure S1.pdf]

Supplementary Figure 2

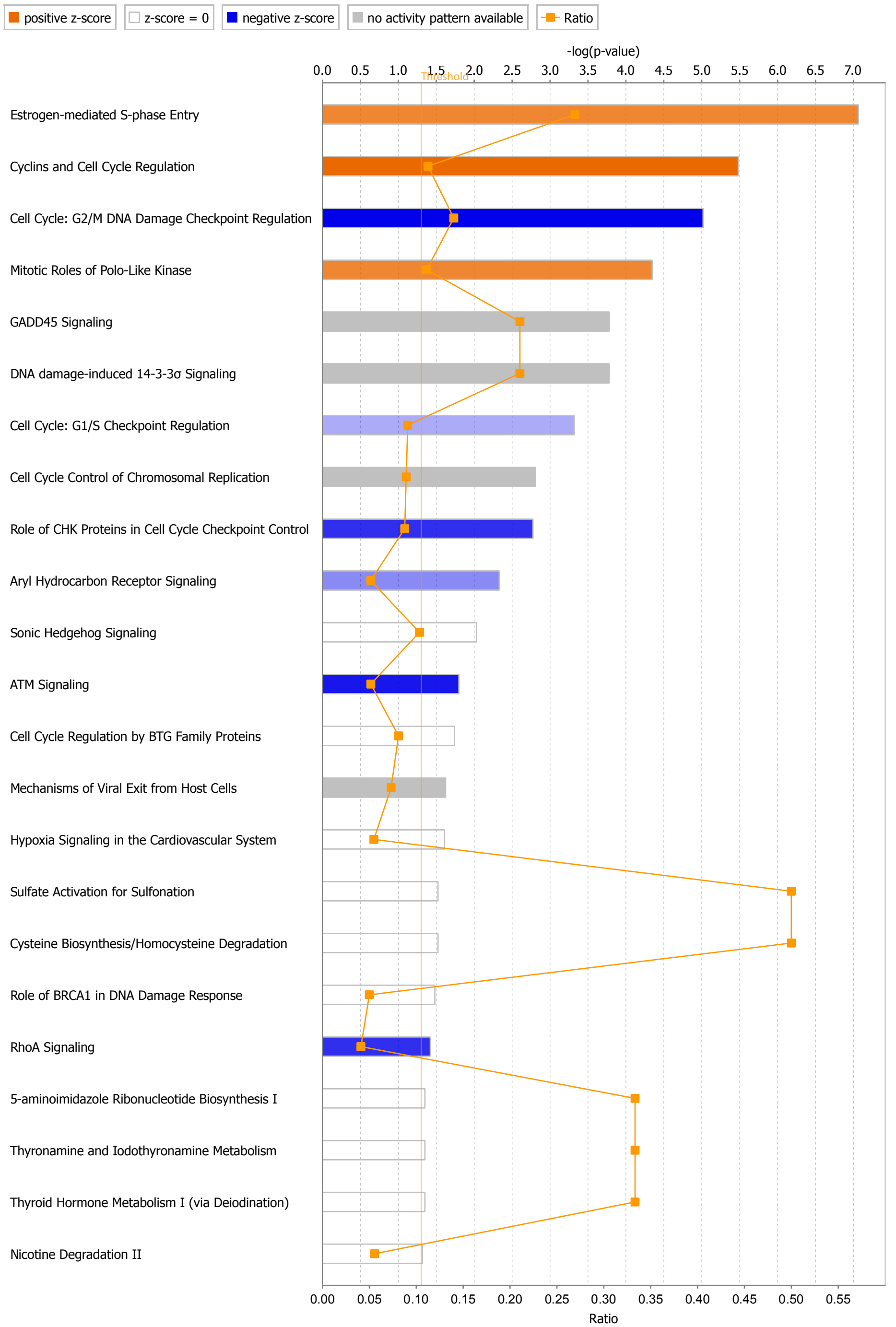

Supplement: Supplementary file 1 [file cancers-11-00882-s001.zip › cancers-520397-Supplementary/Figure S2.pdf]

Supplementary Figure 3A

**miR-30c-5p**

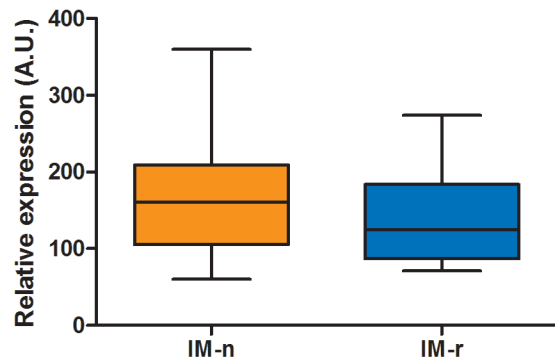

**miR-92a-3p**

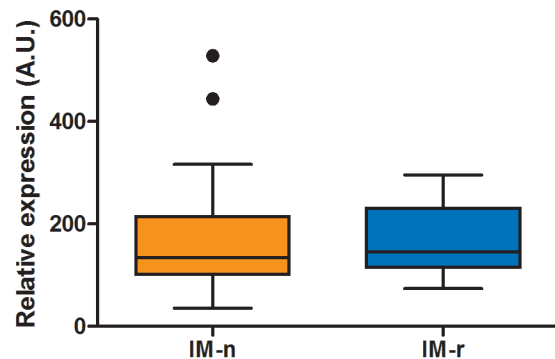

**miR-99a-5p**

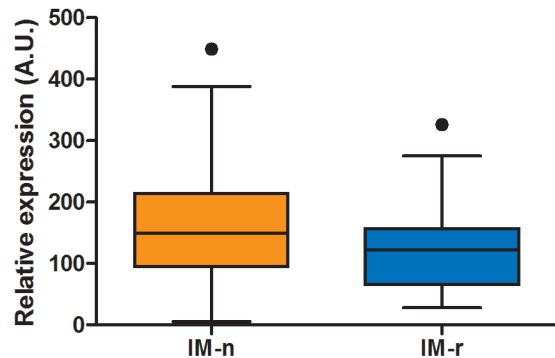

**miR-101-3p**

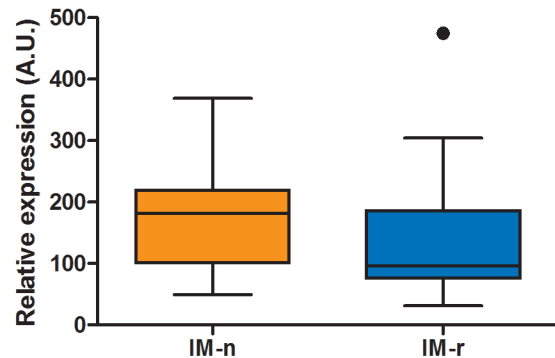

Supplement: Supplementary file 1 [file cancers-11-00882-s001.zip › cancers-520397-Supplementary/Figure S3A.pdf]

Supplementary Figure 3B **AURKA**

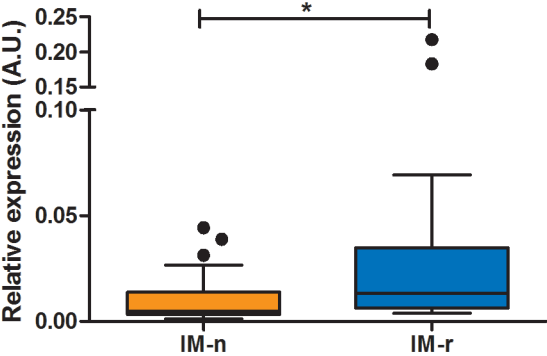

**AURKB**

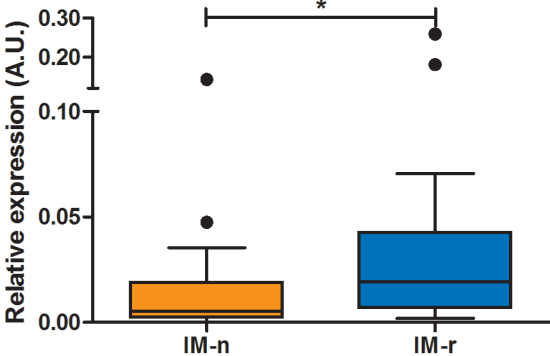

**CCND2**

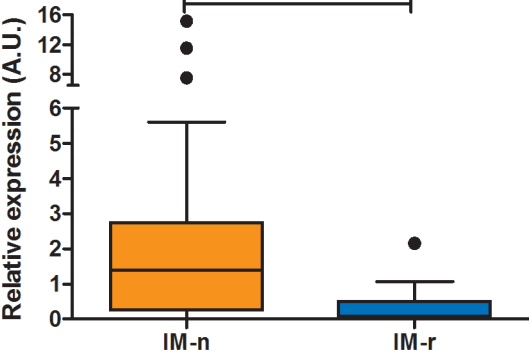

**CCNE2**

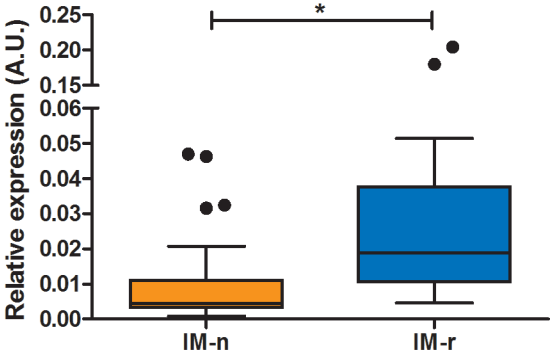

**CDK1**

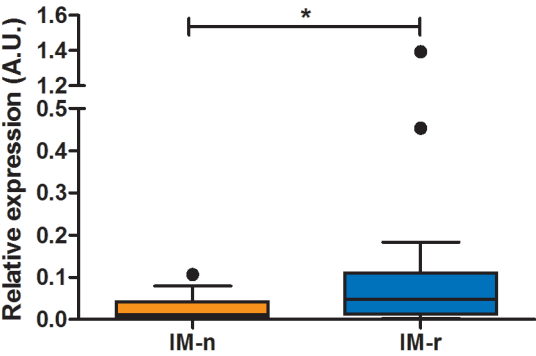

**CDKN1C**

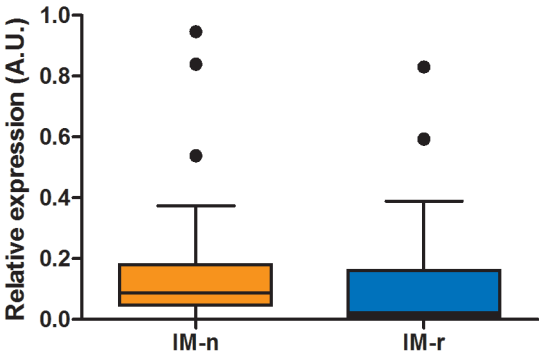

**E2F7**

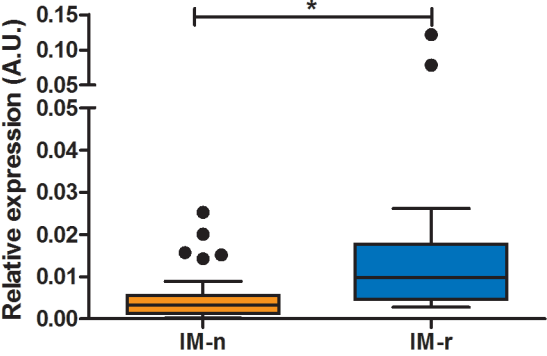

**FOXM1**

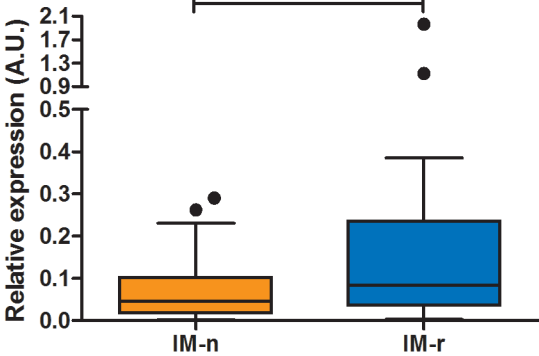

Supplement: Supplementary file 1 [file cancers-11-00882-s001.zip › cancers-520397-Supplementary/Figure S3B.pdf]
